# A game theory-based trust measurement model for social networks

**DOI:** 10.1186/s40649-016-0027-x

**Published:** 2016-05-20

**Authors:** Yingjie Wang, Zhipeng Cai, Guisheng Yin, Yang Gao, Xiangrong Tong, Qilong Han

**Affiliations:** 1 School of Computer and Control Engineering, Yantai University, Qingquan Road, Yantai, 244005 China; 2 School of Mathematics and Information Science, Yantai University, Qingquan Road, Yantai, 244005 China; 3 College of Computer Science and Technology, Harbin Engineering University, Harbin, 150001 China; 4 Department of Computer Science, Georgia State University, Atlanta, 30303 Georgia

**Keywords:** Service reliability, Feedback effectiveness, Recommendation credibility, Game theory, Punishment mechanism

## Abstract

**Background:**

In social networks, trust is a complex social network. Participants in online social networks want to share information and experiences with as many reliable users as possible. However, the modeling of trust is complicated and application dependent. Modeling trust needs to consider interaction history, recommendation, user behaviors and so on. Therefore, modeling trust is an important focus for online social networks.

**Methods:**

We propose a game theory-based trust measurement model for social networks. The trust degree is calculated from three aspects, service reliability, feedback effectiveness, recommendation credibility, to get more accurate result. In addition, to alleviate the free-riding problem, we propose a game theory-based punishment mechanism for specific trust and global trust, respectively.

**Results and conclusions:**

We prove that the proposed trust measurement model is effective. The free-riding problem can be resolved effectively through adding the proposed punishment mechanism.

## Background

With the current popularity of online social networks, more and more information is distributed through social network services [2]. Participants in online social networks want to share information and experiences with as many reliable users as possible [3–5]. Trust is a basis of social network services. However, the modeling of trust is complicated and application-dependent [6–8]. Modeling trust needs to consider interaction history, recommendation, user behaviors and so on. Therefore, modeling trust is an important focus for online social networks [9–11].

In online social websites, such as Amazon, eBay, and FilmTrust, the existing trust models are mainly constructed based on nodes’ global trust. However, these models fail to filter false feedback and distrustful recommendation, which leads to inaccuracy of the measurement results. Because it is common that nodes intend to be selfish, the free-riding phenomenon often occurs in social networks, resulting in the decrease of network performance [12]. For free-riding, the so-called free-riders attempt to benefit from network resources of others without offering their own resources in exchange [13]. The goal of our work is to build an effective trust measurement model that can benefit social network services, such as controlling feedback, recommendation, and strategy selection. In an effort to resolve the above problems, the main contributions of our work are summarized as follows.

1. To more accurately measure trust degree of a node, we introduce three novel evaluation factors which are service reliability, feedback effectiveness and recommendation credibility.
2. Another practical problem considered in this paper is the free-riding problem. We propose punishment mechanisms for specific trust and global trust, respectively, which is different from the existing works where statistic methods are commonly used. In our punishment mechanism, we employ the evolutionary game theory which is more flexible and effective.

The rest of the paper is organized as follows: "Related works" section reviews the related works and presents the motivation for our work. "The proposed trust measurement model" section introduces the proposed trust measurement model for social networks. "Simulations and performance analysis" section illustrates our simulation results and analysis of the results. Conclusions and future work are shown in "The proposed trust measurement model" section.

## Related works

Much effort has been spent on trust measurement models to depict trust behaviors in complex networks. Trust measurement methods under open network environment and trust measurement methods based on Agent synergy are the most important trust measurement methods.

### Trust measurement methods under open network environment

Beth et al. [14] first proposed a trust measurement method under open network environment. In their work, trust is regarded as direct trust and recommendation trust, and a probabilistic method is adopted to represent trust. The PeerTrust model [15] uses the transaction and the community background as the source of reputation feedback. It can act as a defense against some of the subtle malicious attacks, e.g., a seller develops a good reputation by being honest for small transactions and tries to make a big profit by being dishonest for large transactions. The EigenRep model [16] assumes that if the direct trust between a node and the destination node is higher, the recommendation trust is more reliable. The model uses direct trust to calculate the global trust. This model can effectively solve the bad effect caused by the malicious recommendation. Wang et al. [17] proposed a trust model based on Bayesian network. This model investigates how to describe different aspects of trust to obtain various properties of entities according to different scenes. Wang et al. [18] solved the problem of recommendation trust based on the Bayesian method. This method calculates recommendation trust based on experts’ experience. Lu et al. [19] proposed an evaluation method of software reliability. It is a bottom-up calculation process of trust level that can decompose and synthetically derive a parallel structure, so that the trust value of a system can be calculated accurately. However, there are still some shortages about this kind of models. They only adopt probabilistic model to establish subjective trust model. In other words, subjectivity and uncertainty of trust are equivalent to randomness. They also adopt the averaging method to calculate recommendation trust, which cannot reflect the real situations of a trust relationship.

### Trust measurement methods based on Agent synergy

In Agent synergy, trust means that a collaborative agent can properly and nondestructively predict subjective possibility of a collaborative activity. The source of prediction is the goal service behavior that previous agent observes. Prediction results are affected by evaluation of important degree from the agent, such as key collaborative activities and secondary collaborative activities [20, 21]. The eBay trust model is one of the most successful cases. In this model, the entities evaluate each other after each transaction. The structure of this system is straightforward, and the computation cost is small. Because trust between agents is associated with other entities’ subjective understanding and fuzziness, it cannot be described and managed by conventional and accurate logic. Subjective trust as a cognitive phenomenon, whose subjectivity and uncertainty present fuzziness, is often managed by Fuzzy Set-based methods. It not only reflects fuzziness of agent trust, but also describes the trust mechanism between agents with intuitive and concise semantics. Tang et al. [22] first proposed the definition and evaluation of trust based on the fuzzy set theory. They gave formalization and deducted rules of trust to construct a complete subjective trust management model. However, this kind of model fails to consider the cooperative cheating behaviors, which cannot detect the community of cooperative cheating.

In addition, some recent works are also remarkable. Shi et al. [23] proposed a dynamic P2P trust model based on the time-window feedback mechanism. The model considers the inherent connection among trust, reputation and incentive and the effect of time factor on the trust computation. Gan et al. [24] proposed a reputation-based multi-dimensional trust (RMDT) algorithm which makes use of a self-confident coefficient to synthesize the direct and recommendation trust to evaluate the nodes in a network. A multi-dimensional trust mechanism is also introduced to improve sensitivity of RMDT on a single attribute. Meng et al. [25] proposed the @Trust model. Bedi et al. [26] proposed a trust-based recommender system using ant colony for trust computation. Zhang et al. [27] proposed a trust evaluation method based on the cloud model.

These models have promoted the development of trust measurement models. However, most of the existing models failed to filter false feedback and distrustful recommendation, which leads to inaccuracy of measurement results. In addition, free-riding problem was not comprehensively considered in most of the existing trust measurement models. Considering these problems, this paper proposes a game theory-based trust measurement model for social networks. The proposed model introduces three novel evaluation factors which are service reliability, feedback effectiveness and recommendation credibility to more accurately measure the trust degree of a node.

## The proposed trust measurement model

To describe the trust degree more accurately, this paper divides nodes into four categories, which are service nodes, feedback nodes, recommendation nodes and managed nodes. In social networks, *trust* represents the level of confidence about the reliability and correctness of entity’s behaviors. *Service reliability* indicates the trustworthiness of service that service nodes provide; *feedback effectiveness* represents the trustworthiness of feedback that feedback nodes return; *recommendation credibility* expresses the trustworthiness of recommendation that recommendation nodes give. In this paper, the global trust of the node *i*, denoted as $T_i$, is the probability of *i* being correct. The service reliability is denoted as ST$_i$; the feedback effectiveness is denoted as FT$_i$; and the recommendation credibility is denoted as CT$_i$.

In this paper, let *i* be a service node, *j* be a feedback node and *k* be a recommendation node; and $M_i$, $M_j$, $M_k$ are the managed nodes of *i*, *j*, *k*, respectively.

When feedback node *j* requests a specific service *s*, the managed node $M_j$ searches for the trust node which can provide service *s*. If there exists such a node, say node *i*, node *j* requests the service from node *i*. If not, $M_j$ searches for the recommendation node *k*. Then, node *k* recommends a service node *i* with the maximum trust degree that can provide service *s* to node *j*. If there does not exist a recommendation node *k*, the transaction fails.

### The trust measurement process

In this model, the specific feedback value $f_{v_{j,i}}$, given by the feedback node *j*, is known by the system. Therefore, we obtain the calculation method of service reliability based on the specific feedback value $f_{v_{j,i}}$, which is shown by Eq. (1).

1 $$\begin{aligned} \mathrm{ST}_i=\frac{\sum _{j\in \mathrm{set}(i)}^{ }f_{v_{j,i}}\cdot \lambda (j,i) }{\sum _{j\in \mathrm{set}(i)}^{ }{\lambda }(j,i)},\quad \mathrm{FT}_j\ge \theta \end{aligned}$$

In Eq. (1), set(*i*) is the set of feedback nodes that communicated with service node *i*, and $\theta$ is the threshold of feedback effectiveness. ${\lambda }(j,i)$ presents the influence effect of node *j* on node *i*. In addition, FT$_j$ represents the feedback effectiveness of node *j*.

In social networks, some feedback nodes may evaluate some trust nodes maliciously and praise some distrustful nodes. Therefore, we should also evaluate the trust degree of $f_{v_{j,i}}$. In this paper, we calculate the feedback effectiveness based on similarity of specific feedback values. The feedback effectiveness of node *j* can be derived through a similarity formula as shown by Eq. (2).

2 $$\begin{aligned} \mathrm{FT}_j=\frac{\sum _{i\in \mathrm{set}(j,r)}^{ }f_{v_{j,i}}\cdot f_{v_{r,i}}}{\sqrt{\sum _{i\in \mathrm{set}(j,r)}^{ }f^2_{v_{j,i}}}\cdot \sqrt{\sum _{i\in \mathrm{set}(j,r)}^{ }f^2_{v_{r,i}}}} \end{aligned}$$

In Eq. (2), set(*j*, *r*) presents the node-pair set that both nodes communicated with node *i*. Similar with the calculation method of service reliability, the recommendation credibility of node *k* is computed by Eq. (3).

3 $$\begin{aligned} {\text{CT}}_k=\frac{\sum _{i\in \mathrm{Rset}(k)}^{ }{\text{ST}}_i\cdot \lambda (k,i) }{\sum _{i\in \mathrm{Rset}(k)}^{ }{\lambda }(k,i)} \end{aligned}$$

In Eq. (3), Rset(*k*) is the node set recommended by recommendation node *k* before. ${\lambda }(k,i)$ presents the influence effect of node *k* on node *i*. There are two factors affecting the value of ${\lambda }(k,i)$. One is the time interval $T=t_n-t_p$, $t_n$ presents the current time, and $t_p$ presents the time that node *k* recommends node *i*. Another is the connection degree $\omega _{k,i}$ of the relationship between node *i* and node *k*. Thus, ${\lambda }(k,i)$ is shown as Eq. (4).

4 $$\begin{aligned} \lambda (k,i)=\frac{1}{t_n-t_p}\cdot \omega _{k,i} \end{aligned}$$

In this paper, how to determine the connection degree $\omega _{k,i}$ is considered. According to the successful transaction Tr$_\mathrm{suc}$ and the number of total transactions |*Tr*| between node *k* and node *i*, we determine the connection degree $\omega _{k,i}$, which is shown as Eq. (5). In Eq. (5), successful transaction Tr$_\mathrm{suc}$ is an indicative function, if CT$>\mathrm{Threshold}$, Tr$_\mathrm{suc}=1$, otherwise, Tr$_\mathrm{suc}=0$.

5 $$\begin{aligned} \omega _{k,i}=\frac{\sum _{m=1}^{|Tr|}\mathrm{Tr}_\mathrm{suc}}{|Tr|} \end{aligned}$$

According to the above analysis, the global trust degree is shown in Eq. (6). In Eq. (6), $\alpha$, $\beta$ and $\gamma$ are weights for service reliability, feedback effectiveness and recommendation credibility, and $\alpha +\beta +\gamma =1$.

6 $$\begin{aligned} T_i=\alpha \cdot \mathrm{ST}_i+\beta \cdot \mathrm{FT}_i+\gamma \cdot \mathrm{CT}_i \end{aligned}$$

If a service node provides distrust service, i.e. the service reliability is less than the service threshold $\rho$, the node will enter the service punishment cycle. In the service punishment cycle, a node should not provide any service. If a feedback node provides distrust feedback, i.e. the feedback effectiveness is less than the feedback threshold $\theta$, the node will enter the feedback punishment cycle. In the feedback punishment cycle, a node should not request any service. If a recommendation node provides distrust recommendation, i.e. the recommendation credibility is less than the recommendation threshold $\delta$, the node will enter the recommendation punishment cycle. In the recommendation punishment cycle, a node should not provide any recommendation.

The process of direct interaction is summarized in Algorithm 1. In this direct interaction algorithm, the service reliability ST$_i$ and the feedback effectiveness FT$_j$ will be output.

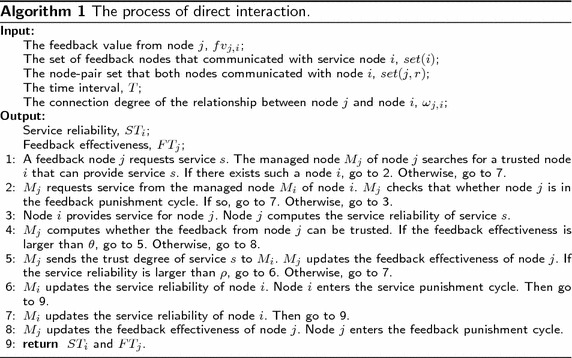



If there is not a trusted service node *i* that has interacted with the feedback node *j* directly, it needs a recommendation node *k* to recommend a trusted node *j* for service node *i*. Thus, the process of indirect interaction is summarized in Algorithm 2.

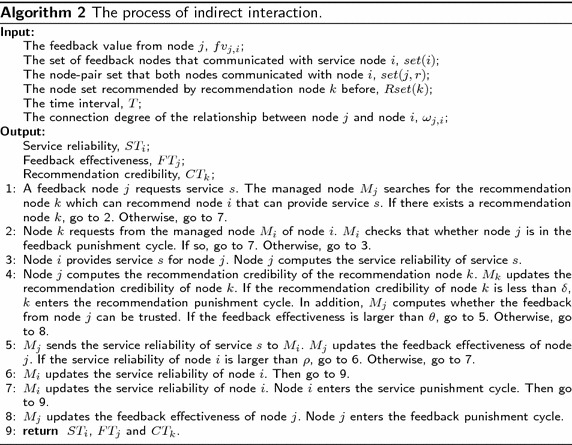



### The punishment mechanisms

To resolve free-riding problem in social networks, two punishment mechanisms are proposed for specific trust and global trust degree, respectively. According to specific trust (service reliability, feedback effectiveness and recommendation credibility), this paper designs three punishment cycles, so that to restrain the specific trust behaviors of nodes. According to global trust, this paper gives a game theory-based punishment mechanism [28] to resolve the free-riding problem for social networks.

For specific trust, we design a specific punishment mechanism and divide punishment cycles into service punishment cycle, feedback punishment cycle and recommendation punishment cycle. Once a node has selfish behaviors, the node will enter punishment cycle. In the period of punishment cycle, the node must be cooperative and honest to restore its reputation. In addition, other nodes reject to provide services to this node. After the punishment cycle, the node can replay transactions. According to different selfish behaviors, this paper gives different punishment strategies, which are shown as followings.

1. Service punishment cycle. If the service reliability ST$_i<\rho$, node *i* will enter service punishment cycle. In the service punishment cycle, a node cannot provide service for other nodes and cannot request any service.
2. Feedback punishment cycle. If the feedback effectiveness FT$_i<\theta$, node *i* will enter feedback punishment cycle. In the feedback punishment cycle, a node cannot request any service. However, it can provide service for other nodes.
3. Recommendation punishment cycle. If the recommendation credibility CT$_i<\delta$, node *i* will enter recommendation punishment cycle. In the recommendation punishment cycle, a node cannot recommend any node. However, it can request and provide service for other nodes.

According to global trust, this paper proposes a punishment mechanism based on multi-strategy game to inspire nodes to select the strategies with high trust degree. T$_i$ indicates the whole trust degree of node *i*. We divide trust degrees into five levels as shown in Table 1.

**Table 1:** The division of trust levels

T_i_	Trust
[0.8,1]	Trust 1
[0.6,0.8)	Trust 2
[0.4,0.6)	Trust 3
[0.2,0.4)	Trust 4
[0,0.2)	Trust 5

The five-strategy matrix is shown in Table 2. In Table 2, pr$_{A}^{ij}$ is the profit value that node *A* obtains, if *A* game with *B* that *A* adopts strategy *i*, and entity *B* adopts strategy *j*. And pr$_{B}^{ij}$ is the profit value that *B* obtains, if *B* game with *A* that *B* adopts strategy *i*, and *A* adopts strategy *j*. Through game analyzing nodes’ behaviors in social networks, we can know that the multi-strategy game matrix is a symmetric matrix. In the analysis for dynamics model, this game is performed repeatedly. At the end of each stage of multi-strategy game, any participant’s strategy as a historical information can be known by other participants. In addition, all participants select and update their strategies for next stage of game based on historical information.

**Table 2:** The initial five-strategy game matrix

Trust level	Trust 1	Trust 2	Trust 3	Trust 4	Trust 5
Trust 1	pr$_{A}^{11},\mathrm{pr}_{B}^{11}$	pr$_{A}^{12},\mathrm{pr}_{B}^{12}$	pr$_{A}^{13},\mathrm{pr}_{B}^{13}$	pr$_{A}^{14},\mathrm{pr}_{B}^{14}$	pr$_{A}^{15},\mathrm{pr}_{B}^{15}$
Trust 2	pr$_{A}^{21},\mathrm{pr}_{B}^{21}$	pr$_{A}^{22},\mathrm{pr}_{B}^{22}$	pr$_{A}^{23},\mathrm{pr}_{B}^{23}$	pr$_{A}^{24},\mathrm{pr}_{B}^{24}$	pr$_{A}^{25},\mathrm{pr}_{B}^{25}$
Trust 3	pr$_{A}^{31},\mathrm{pr}_{B}^{31}$	pr$_{A}^{32},\mathrm{pr}_{B}^{32}$	pr$_{A}^{33},\mathrm{pr}_{B}^{33}$	pr$_{A}^{34},\mathrm{pr}_{B}^{34}$	pr$_{A}^{35},\mathrm{pr}_{B}^{35}$
Trust 4	pr$_{A}^{41},\mathrm{pr}_{B}^{41}$	pr$_{A}^{42},\mathrm{pr}_{B}^{42}$	pr$_{A}^{43},\mathrm{pr}_{B}^{43}$	pr$_{A}^{44},\mathrm{pr}_{B}^{44}$	pr$_{A}^{45},\mathrm{pr}_{B}^{45}$
Trust 5	pr$_{A}^{51},\mathrm{pr}_{B}^{51}$	pr$_{A}^{52},\mathrm{pr}_{B}^{52}$	pr$_{A}^{53},\mathrm{pr}_{B}^{53}$	pr$_{A}^{54},\mathrm{pr}_{B}^{54}$	pr$_{A}^{55},\mathrm{pr}_{B}^{55}$

To prevent selfish nodes from selecting the strategy with low trust degree to be their preferred strategy for getting more benefits, i.e. to restrain the free-riding phenomenon, a punishment mechanism is established to inspire nodes to select the strategies with high trust degree based on the multi-strategy game. In the case of $i<j$, the calculation method of benefits after adding punishment mechanism is shown by Eq. (7). When $i=j$, the calculation method is shown by Eq. (8).

7 $$\begin{aligned} \begin{array}{l} \mathrm{PR}_A^{ij} = \mathrm{pr}_A^{ij} + \mu \cdot (\mathrm{pr}_A^{ij} + \mathrm{pr}_B^{ij}), \begin{array}{*{20}{c}} {}&{}{i < j} \end{array}\\ \mathrm{PR}_B^{ij} = \mathrm{pr}_B^{ij} - \mu \cdot (\mathrm{pr}_A^{ij} + \mathrm{pr}_B^{ij}) \end{array} \end{aligned}$$

8 $$\begin{aligned} \mathrm{PR}_A^{ij} = \mathrm{pr}_A^{ij},\begin{array}{*{20}{c}} {}&{i = j} \end{array} \end{aligned}$$

When two nodes game with each other, for the node with higher trust degree, system will increase its rewards; for the node with lower trust degree, system will decrease its earnings. $\mu$ is a punishment parameter. Because the game matrix is a symmetric matrix, the punishment parameter is symmetric too. In this paper, if the value of (Trust *i* $-$ Trust *j*) is bigger, $\mu$ will increase.

### Complexity analysis

In this trust measurement model, we need to compute service reliability, feedback effectiveness and recommendation credibility for one node. According to a node’s service reliability, the computational complexity is *O*(*l*), where *l* represents that this node has provided services for *l* nodes ever before. According to a node’s feedback effectiveness, the computational complexity is *O*(*m*), where *m* indicates that *m* nodes provided feedbacks for this node. According to a node’s recommendation credibility, the computational complexity is *O*(*n*), where *n* represents that *n* nodes were recommended by this node before. Therefore, the computational complexity of a node’s global trust is $O(l+m+n)$. The proposed trust measurement model can be computed in polynomial time, thus it is computationally efficient.

## Simulations and performance analysis

In this section, we present the simulation results to verify the effectiveness of the proposed model. The hardware simulation environment is: Intel Core (TM) Duo 2.66 GHz CPU, 2GB Memory, Windows XP operating system, and Matlab 7.0 simulation platform. We simulate real online social networks in our experiments. In simulations, 1000 nodes are simulated. There are two kinds of nodes, normal nodes and malicious nodes. There are two types of normal nodes which are completely trustful nodes that can provide trustful service, feedback and recommendation, and mix-type trustful nodes that provide trustful feedback and recommendation, but random service quality. 20 % of files have low quality, i.e. malicious files. Malicious nodes include three types which are completely malicious nodes that provide questionable service, feedback and recommendation, random malicious nodes that provide questionable service, feedback and recommendation with a certain probability (in the simulations, the probability is 50 %), and disguised malicious nodes that provide trustful service and recommendation but questionable feedback. In the simulations, there are 1000 nodes, including 30 % completely trustful nodes, 30 % mix-type trustful nodes, 10 % completely malicious nodes, 20 % random malicious nodes, and 10 % disguised malicious nodes. The simulation setting is shown in Table 3 where 1 represents completely trustful, 0 represents completely questionable, and $\varepsilon$ represents randomly trustful.

**Table 3:** The simulation setting

The style of nodes/trust	Service	Feedback	Recommendation
Ct	1	1	1
Tt	$\varepsilon$	1	1
Cm	0	0	0
Rm	$\varepsilon$	$\varepsilon$	$\varepsilon$
Dm	1	0	1

### Experimental verification for specific trust

**Fig. 1:**
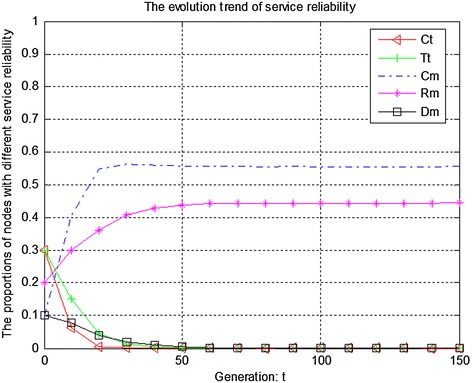
The initial evolution trend of service reliability without punishment

**Fig. 2:**
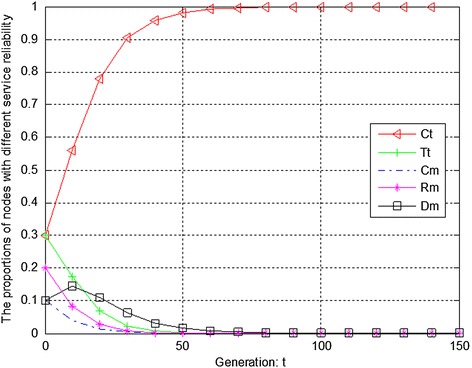
The evolution trend of service reliability with punishment

**Fig. 3:**
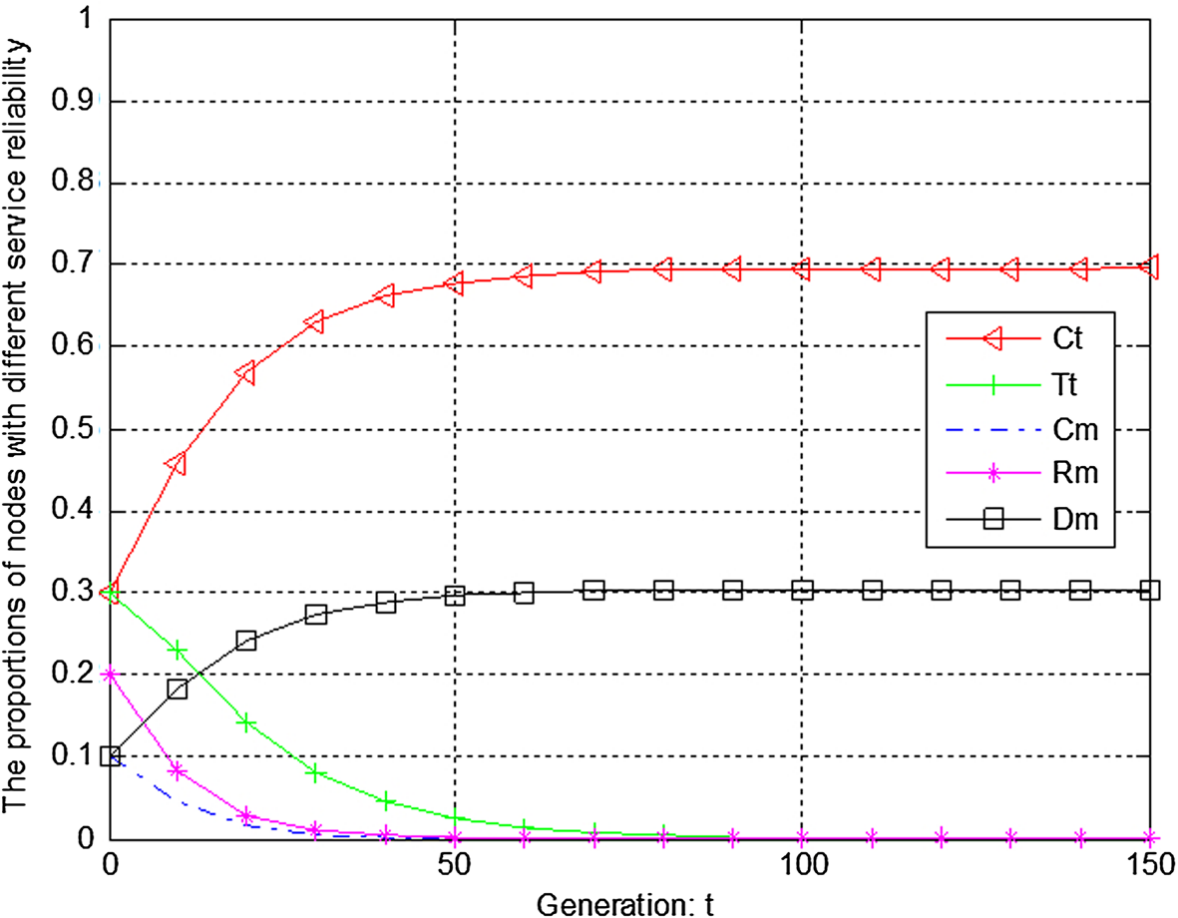
The evolution trend of service reliability with adjusted punishment

We measure the evolution of trust degree according to service reliability, feedback effectiveness and recommendation credibility respectively. Figure 1 presents the initial evolution trend of service reliability without any punishment mechanism. From Fig. 1, it can be seen that there exists free-riding problem. The proportions of completely malicious nodes (Cm) and random malicious nodes (Rm) increase steadily in first 50 generations. After that, network tends to be stable. Therefore, if there is not any punishment mechanism, malicious nodes will dominate the evolutionary direction of the whole network. Figure 2 shows the ideal condition by adopting punishment mechanism. From Fig. 2, it can be seen that completely trustful nodes (Ct) will dominant the evolutionary direction of the whole network with the proposed punishment mechanism. However, the proportion of any other type of nodes will decrease to 0. In this case, only completely trustful nodes (Ct) can survive in network. It will cause new nodes entering network to be dead, because they do not have any historical trust information. To resolve this problem, we adjust the strength of punishment to avoid cold boot problem, as shown in Fig. 3. From Fig. 3, it can be seen that the proportions of completely trustful nodes (Ct) and disguised malicious nodes (Dm) increase steadily, and tend to be stable in the end. It is because that disguised malicious nodes (Dm) can provide trustful service so that they can survive in network.

**Fig. 4:**
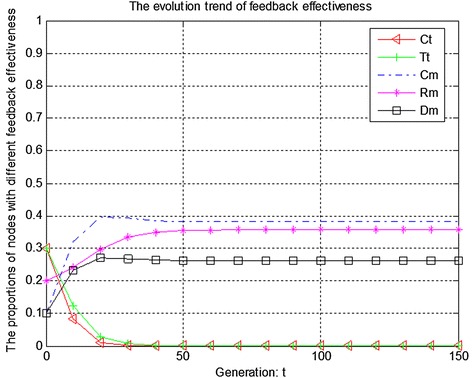
The initial evolution trend of feedback effectiveness without punishment

**Fig. 5:**
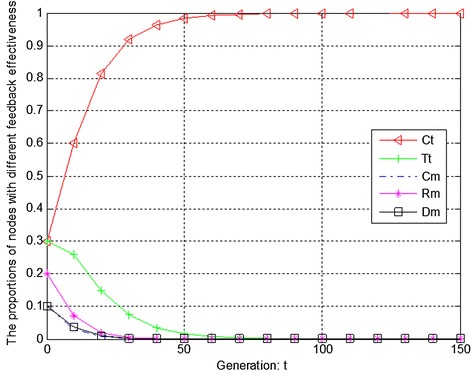
The evolution trend of feedback effectiveness with punishment

**Fig. 6:**
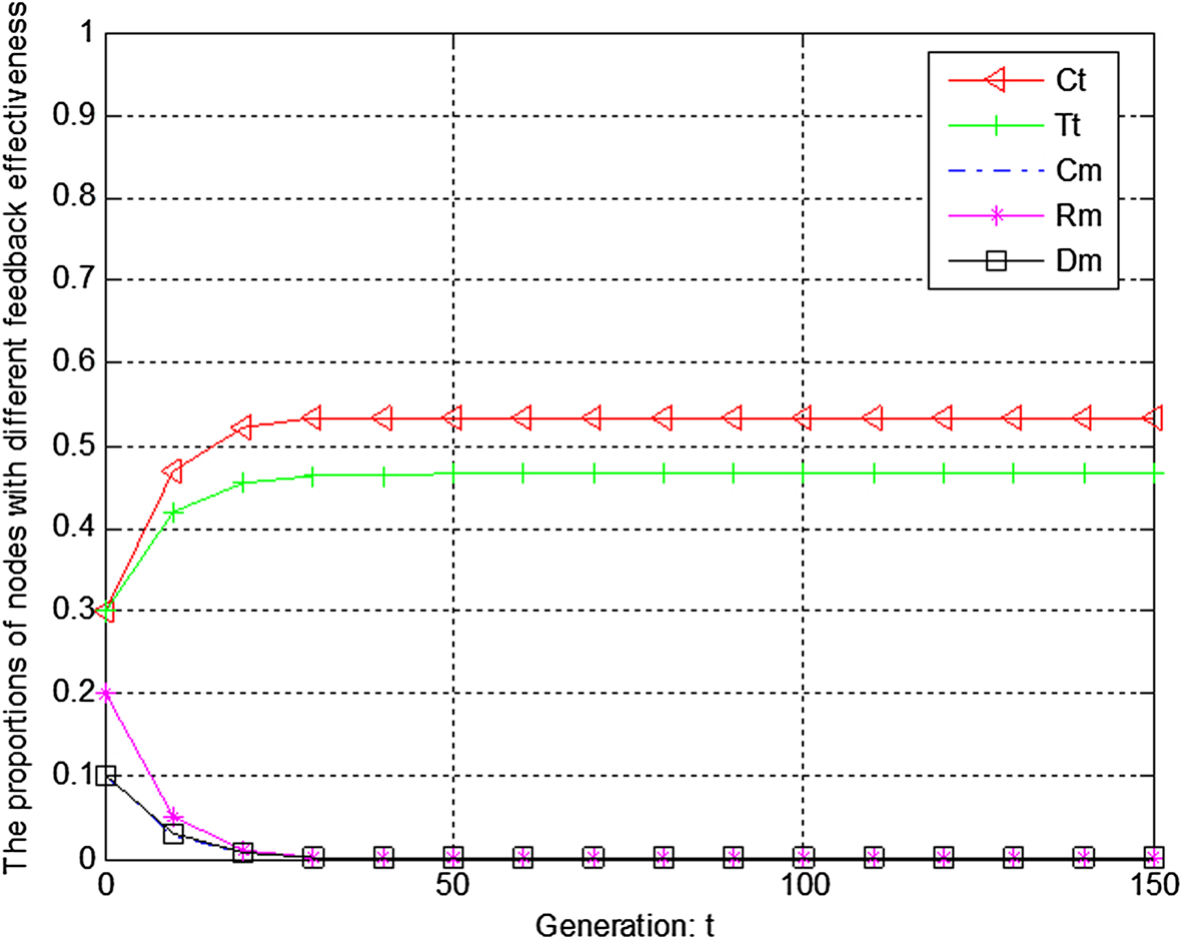
The evolution trend of feedback effectiveness with adjusted punishment

Figures 4, 5 and 6 show the evolution trend of feedback effectiveness. Figure 4 presents the initial evolution trend of feedback effectiveness without any punishment mechanism. From Fig. 4, it can be seen that the free-riding problem occurs. The proportions of completely malicious nodes (Cm), random malicious nodes (Rm) and disguised malicious nodes (Dm) increase steadily in first 50 generations. After that, network tends to be stable. Since disguised malicious nodes (Dm) provide distrustful feedback, they will obtain more benefits than the nodes that provide trustful feedback without any punishment mechanism. Figure 5 shows the ideal case by adopting punishment mechanism. From Fig. 5, it can be seen that completely trustful nodes (Ct) will dominate the evolutionary direction of the whole network with the proposed punishment mechanism. However, the proportion of any other type of nodes will decrease to 0. In this case, only completely trustful nodes (Ct) can survive in network. Figure 6 shows the evolutionary results after adjusting the strength of punishment. It can be seen that the proportions of completely trustful nodes (Ct) and mix-type trustful nodes (Tt) increase steadily, and tend to be stable in the end. This is because that mix-type trustful nodes (Tt) can provide trustful feedback, and they will survive in network.

**Fig. 7:**
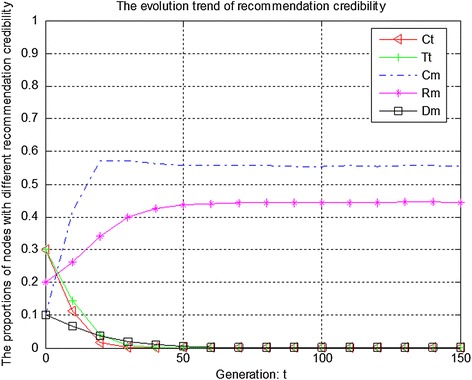
The initial evolution trend of recommendation credibility without punishment

**Fig. 8:**
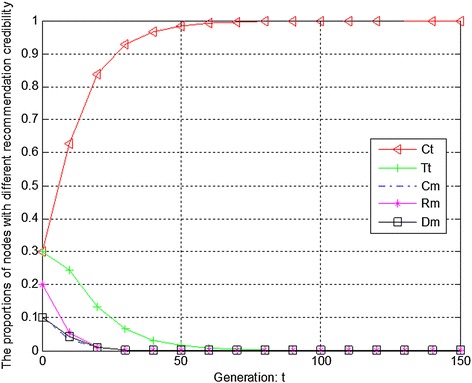
The evolution trend of recommendation credibility with punishment

**Fig. 9:**
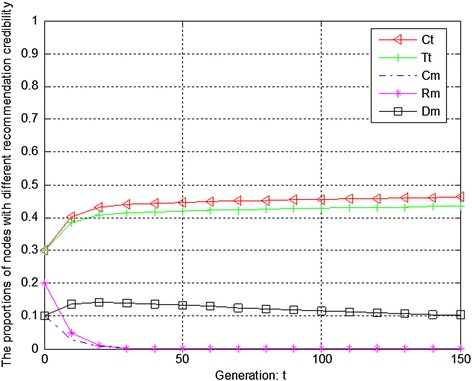
The evolution trend of recommendation credibility with adjusted punishment

Figures 7, 8 and 9 show the evolution trend of recommendation credibility. Figure 7 presents the initial evolution trend of recommendation credibility without any punishment mechanism. From Fig. 7, it can be seen that there is free-riding problem in network. The proportions of completely malicious nodes (Cm) and random malicious nodes (Rm) increase steadily in first 50 generations. After that, network tends to be stable. Figure 8 shows the ideal case by adopting punishment mechanism. From Fig. 8, it can be seen that completely trustful nodes (Ct) will dominate the evolutionary direction of the whole network with the proposed punishment mechanism. However, the proportion of any other type of nodes will decrease to 0. In this case, only completely trustful nodes (Ct) can survive in network. Therefore, Fig. 9 shows the evolutionary results after adjusting the strength of punishment. It can be seen that the proportions of completely trustful nodes (Ct) and mix-type trustful nodes (Tt) increase steadily, and tend to be stable in the end. The reason is that mix-type trustful nodes (Tt) can provide trustful recommendation, and they will survive in network.

### Experimental verification for global trust

**Fig. 10:**
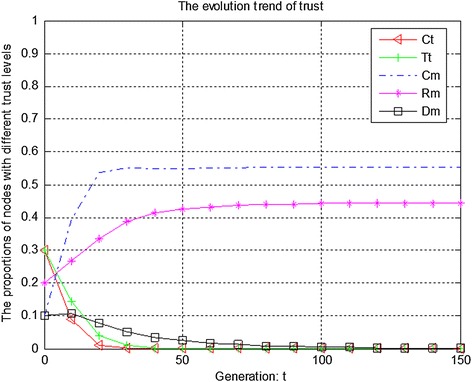
The initial evolution trend of trust without punishment

**Fig. 11:**
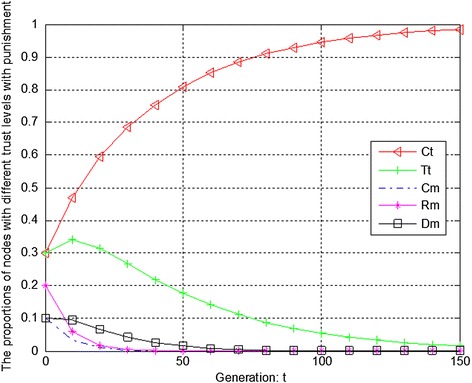
The evolution trend of trust with punishment

We also verify the effectiveness for global punishment mechanism. According to the simulations for specific trust, this section combines the measurement results of service reliability, feedback effectiveness and recommendation credibility to measure the trust evolution of the whole network. Figure 10 shows the initial evolution results. From Fig. 10, we can see that if there is not any punishment mechanism, the free-riding phenomenon will occur. The free-riding problem can be resolved by employing global punishment mechanism as shown in Fig.11.

## Conclusion

In social networks, trust relationships between nodes are the basis of service transactions. However, the establishment of trust relationship is a complex progressive process depending on interaction history, trust recommendation, trust management and so on. Therefore, modeling trust relationship needs to take into account multiple decision factors. Considering the existing problems of trust models, this paper proposes a game theory-based trust measurement model for social networks where trust degree is determined by three aspects, which are service reliability, feedback effectiveness, and recommendation credibility. Based on game theory, we propose punishment mechanisms according to specific trust and global trust respectively to resolve free-riding problem. The simulation results show the effectiveness of the proposed trust measurement model. It also shows that the proposed punishment mechanisms can prevent free-riding phenomenon effectively. As a future work, we will further investigate more specific trust relationships between nodes, e.g., family, best friends, and classmates. We plan to study how to find ordered trust node set in social networks.
